# Organizational justice and organizational citizenship behavior: the mediating role of work engagement

**DOI:** 10.1016/j.heliyon.2022.e09450

**Published:** 2022-05-16

**Authors:** Md. H Asibur Rahman, Dewan Niamul Karim

**Affiliations:** a Department of Business Administration-General, Faculty of Business Studies, Bangladesh University of Professionals, Bangladesh; b Department of Management Studies, Faculty of Business Studies, Jahangirnagar University, Bangladesh

**Keywords:** Procedural justice, Organizational citizenship behavior, Informational justice, Work engagement, Interactional justice, University faculty, Private university

## Abstract

The intention of this paper is to investigate the mediating role of work engagement between the four dimensions of organizational justice (OJ) and organizational citizenship behavior (OCB) dimensions. The current literature appears to have overlooked the mediating role of work engagement (WE) in the link between justice and organizational citizenship behavior (OCB) in higher education institutions (HEIs). As a result, it appears to be one of the primary studies of its kind to investigate the relationship between OJ dimensions (e.g., procedural, distributive, informational, and interpersonal justice) and OCB dimensions (e.g., OCBI and OCBO). Data were collected from 121 faculty members from ten private universities operating in Bangladesh. The PLS-SEM was employed to investigate hypotheses. The investigation found OJ is positively connected to WE. Besides, WE is significantly related to OCB. Moreover, WE mediated the relationship between OJ dimensions and OCB dimensions, except for the relationship between PJ and OCB dimensions. The study provides necessary guidelines for the organizations regarding how they might improve citizenship behavior by ensuring justice and engagement in the workplace. Therefore, the application of the findings might ensure better employee outcomes and organizational productivity.

## 1 Introduction

Higher education institutions (HEIs) in Bangladesh are facing severe competition for survival and success due to the rapid expansion of the sector over the last couple of decades (Karim, 2019). Moreover, these institutions are frequently alleged to have inadequate research activities, poor teaching quality, and an inferior academic environment (Karim and Majid, 2017). Therefore, promoting a culture of learning and knowledge sharing, collaborative research, innovation, and academic excellence is essential for the success and growth of these institutions. It is usually believed that such a culture cannot be promoted without a greater degree of constructive extra-role behaviors from academics. The extra-role behaviors, commonly known as organizational citizenship behavior (OCB), exhibited by the academic staff are believed to be crucial for academic and institutional success (Donglong et al., 2020) and can be a great source of sustainable competitive advantage for any HEI (Butt et al., 2020). Generally, OCB encompasses discretionary and beneficial extra-role behaviors of the an organization's members, which greatly contribute to a variety of desirable work outcomes and the effective functioning of an organization (Polat, 2009; Thompson et al., 2020; Yaakobi and Weisberg, 2020).

OCBs have a variety of impacts on organizational performance, the majority of which are constructive (Tefera and Hunsaker, 2020). Organ (1988) postulated five dimensions of OCB: altruism, courtesy, conscientiousness, civic virtue, and sportsmanship. These five dimensions of OCB are further categorized into two, i.e., OCBI-OCB directed toward specific individuals and OCBO-OCB directed toward the organization (Butt et al., 2020; Williams and Anderson, 1991). Besides, the literature also recognizes these two broad categories of OCB of faculties (Somech, 2016); OCBI-OCB directed toward specific individuals in school; and OCBO-OCB directed toward the organization as a whole (OCBO) (Majeed and Jamshed, 2021; Sesen and Basim, 2012; Williams and Anderson, 1991). The faculty's OCBI includes behaviors that benefit specific individuals, such as students or colleagues (Ho and Le, 2020). For instance, OCBI of teachers includes staying at the office after working hours to share course materials with students (Sesen and Basim, 2012; Somech, 2016), cooperating with colleagues and supervisors in their interests (Tan et al., 2019). OCBO includes behaviors that benefit the university (Majeed and Jamshed, 2021), for example, volunteering for jobs that are not compensated and giving inventive ideas to improve the school (Somech, 2016). Besides, OCBO also includes sending advance notice before being unable to come to work, respecting the informal rules, or setting out to maintain order (Ho and Le, 2020; Williams and Anderson, 1991). However, some studies identified three dimensions of OCB, including OCB directed towards schools or universities as a whole, OCB toward colleagues/team, and OCB toward students (Ahmed, 2021; Belogolovsky and Somech, 2012).

Thus, faculty members OCBs include, for example, supporting other faculty members with job pressure (Halid et al., 2020); giving more detailed descriptions and examples to pupils (Donglong et al., 2020); taking care of new faculty members to cope up (Halid et al., 2020); proposing reforms and so on plays a very significant role as these unpaid operations validate additional university services (Runhaar et al., 2013). Additionally, they are more tolerant of minor inconveniences, more cooperative at work, and more engaged in employee social organizations (Halid et al., 2020). Thus, OCBs may work as critical drivers of faculty efficiency and productivity in the education sector and they have a positive impact on the overall performance of higher educational institutions (Dirican and Erdil, 2016; Halid et al., 2020). However, prior OCB researchers have mostly focused on the context of business organizations, whereas only limited studies have attempted to explore the determining factors of OCBs of academic staff in the higher education setting (Ababneh and Hackett, 2019; Donglong et al., 2020).

In the HEIs, those limited studies explored several important determinants of OCBs, such as include inclusive leadership (Aboramadan et al., 2021), organizational identification (Sidorenkov and Borokhovski, 2021; Wilkins et al., 2018), job satisfaction (Dong and Phuong, 2018; Wilkins et al., 2018), leadership self-efficacy (Adewale and Ghavifekr, 2019), organizational commitment (Claudia, 2018), affective commitment (Donglong et al., 2020), transformational leadership (Majeed et al., 2017; Masood et al., 2020; Nasra and Heilbrunn, 2016), spiritual leadership (Djaelani et al., 2021), organizational support (Rosafizah et al., 2020), organizational politics (Atta and Khan, 2016), and organizational justice (Awang and Ahmad, 2015; Dong and Phuong, 2018; Donglong et al., 2020; Junru and Huang, 2019; Tran, 2016). Moreover, organizational justice (OJ) is a significant predictor of OCB (Colquitt et al., 2013; Khaola and Rambe, 2020; Tefera and Hunsaker, 2020). In a specific context, "justice" refers to the legality, equitability, and relevance of an action or decision (Donglong et al., 2020). Therefore, there is more or less consensus on the idea that organizational justice (OJ) is a strong determinant of OCBs in academic settings. Specifically, a variety of research has been done in different commercial organizations based on the relationship between the perception of OJ and OCB (Ali, 2016; Chan and Lai, 2017; Donglong et al., 2020; Lim and Loosemore, 2017). Following a thorough and exhaustive review of the previous literature, it was observed that, with few exceptions (see, Donglong et al., 2020; Sujono et al., 2020), little is known regarding the mechanism through which OJ can stimulate academic staff's involvement in OCB is still unexplored in the context of HEIs.

In other words, fairness in the organization does not lead directly to OCB. Prior studies explored several intervening variables between OJ and OCB, such as organizational commitment (Donglong et al., 2020; Jehanzeb and Mohanty, 2019), organizational trust (Ullah et al., 2021), job satisfaction (Junru and Huang, 2019; Khan et al., 2020), job involvement (Sujono et al., 2020), psychological ownership (Jnaneswar and Ranjit, 2021), and leader-member exchange (Hasyim and Palupiningdyah, 2021; Sheeraz et al., 2020). However, there is still a call to uncover the “black box” remaining in the OJ—OCB relation (Ambrose et al., 2021; Rana et al., 2018; Zayed et al., 2020). Considering the role of OJ on employee attitude and behavior, very few studies have explored the mediating role of work engagement (WE), a favourable employee attitude, in the association between organisational justice and OCB across the sectors (Hassan et al., 2014; Yanthi and Rahyuda, 2019). To fill up the gap, the study proposes work engagement, a favorable work attitude, as a probable mediating mechanism between perceived organizational justice and the OCBs of academic staff. WE is defined as “a positive, fulfilling, work-related state of mind that is characterized by vigor, dedication, and absorption” (Schaufeli et al., 2002, p. 72). Where vigor relates to high-level activation; in the meantime, dedication entails escalated identification, and absorption implies full concentration (Han et al., 2021). Applying Social Exchange Theory (SET) (Blau, 1964; Cropanzano and Mitchell, 2005) it is possible to establish a link among the OJ, WE, and OCB. The theory suggests that if employees perceive that they are fairly treated in the organization, they will reciprocate it with a favorable attitude like WE, which will result in adopting voluntary behaviors helpful to the organizations and their employees (Hassan et al., 2014). Therefore, the intervening role of WE in the link between OJ and OCB in the context of the higher education sector can be explored. Moreover, it is crucial to conduct empirical research on how different kinds of organisational justice affect employees' attitudes and behaviours (Jang et al., 2021). Therefore, this research will endeavour to fill up the gap left in the previous studies and explore the mediating role of WE in the relationship of OJ and OCB in the context of HEIs in Bangladesh.

Like most of the countries, universities in Bangladesh belong to both public and private sector. However, due to various influencing factors, we have opted to conduct the study in the private higher education institution context. Targeting private universities instead of public universities is more appropriate in relation to studying HR policies and practices. The reason is that HR policies and practices in all public universities are almost the same and are regulated by the government whilst they differ in private universities (Karim, 2019). The policymakers and governors of public universities in Bangladesh are the Vice-Chancellor, Pro-Vice-Chancellor, Treasurer, and Syndicate (highest decision-making body) and Senate (budget-approving body) members. Except for the Senate, private universities have all the above. Traditionally, the chairman and the Board of Trustees perform the role of the Senate and have the unlimited ability to influence private university governance in whatever way they want (Sarkar and Hossain, 2019). Therefore, there is a continuous crisis of justice in terms of distribution of resources, organizational procedures and interaction with the faculties and staff. In particular, the HRM practices (compensation and salary packages) in public universities are uniform and equally applicable to all. In contrast, compensation and salary packages in private universities are highly diverse (Karim, 2019) and designed in a customized manner to suit the management's best interests. Besides, the chairman and board of trustees' autocracy and the poor performance of these universities have been the subject of regular reporting in national newspapers in recent times.

## 2 Literature review and hypotheses development

### 2.1 Organizational justice and work engagement

The conception of justice as part of an academic practice has evolved over 2,000 years ago (Stamenkovic et al., 2018). Justice refers to whether an action or decision is legally fair and considered to be relevant to the context of a decision (Donglong et al., 2020). There are three primary types of justice: i) distributive justice (DJ), ii) procedural justice (PJ), and iii) interactional justice (IJ) (Ahmed et al., 2018; Lee and Chui, 2019; Stamenkovic et al., 2018). In addition, several other researchers have identified four dimensions of OJ—i) DJ, ii) PJ, ii) informational justice (Inf.J), and iv) interpersonal justice (Int.J) (Fortin et al., 2020; Sharma and Kumra, 2020). The principle of DJ is drawn from the equity theory (Stamenkovic et al., 2018), which illustrates how individuals compare their results (rewards) to inputs (knowledge, skills, and abilities) relative to other people (Adams, 1965; Donglong et al., 2020; Fortin et al., 2020; Stamenkovic et al., 2018). In addition, PJ is related to the perceived fairness and appropriateness of individuals in the decision-making process in organizations (Ahmed et al., 2018; Donglong et al., 2020; Fortin et al., 2020), including how clear and reliable results are implemented, the capacity to voice during the process, ethical and impartial decision-making or lack of prejudice, and correctness (Ahmed et al., 2018). Therefore, PJ put importance on the impartiality of the policies and procedures through which outcomes are determined (Chan and Lai, 2017; Donglong et al., 2020). Int.J refers to the degree to which workers are treated properly and respectfully within an institution (Ahmed et al., 2018; Donglong et al., 2020; Sharma and Kumra, 2020). In addition, the Int.J is related to the care of persons with courtesy, honesty and respect by superiors in the process (Chan and Lai, 2017; Sharma and Kumra, 2020). Inf.J applies to the degree to which the clarifications given are aligned with decisions made in an institution (Donglong et al., 2020). Therefore, Inf.J exists when (i) Adequate knowledge and clarity on the decisions made (Lim and Loosemore, 2017); (ii) people participate in decision-making processes (Cohen-Charash and Spector, 2001; Lim and Loosemore, 2017); and (iii) communications shall be rendered in a timely and clear manner (Chan and Lai, 2017; Lim and Loosemore, 2017; Schumacher et al., 2020).

Employees perceived OJ shall affect their approach to their job (Jang et al., 2021; Saks, 2006). In addition, workers' understanding of justice in the work environment produces a perception of optimistic self-image and self-identity that eventually serves as a powerful catalyst to commitment (Park et al., 2016). Kahn's (1990) seminal work defined WE as "the simultaneous employment and expression of a person's 'preferred self' in task behaviors that promote connections to work and to others, personal presence behaviors that promote connections to work and to others, personal presence (physical, cognitive, emotional), and active, full role performances” (p. 700). WE is often described as an active state of well-being at job that is marked by vigor, commitment and absorption (Ahmad and Gao, 2018; Kaski and Kinnunen, 2021; Wood et al., 2020). In comparison, people are more indebted because they have a high feeling of fairness in their companies and are therefore fair to play their tasks with a higher degree of dedication (Donglong et al., 2020; Schumacher et al., 2020).

OJ predicts positive workplace outcomes (Matteson et al., 2021). When organizations ensure just and fair treatment, courteous interaction, ethical and impartial decision-making, and equitable benefit and compensation packages, they share necessary information and feedback with employees. In return, employees would like to demonstrate positive work-related outcomes for the behavior and actions they received. One reason for such positive outcomes might be social exchange relations as per the SET (Blau, 1964). SET says that when two parties have a beneficial interaction in a reciprocal relationship, the benefits don't have to be economic in either case. They can be based on experience and the development of positive attitudes and behaviors between the two parties (Farid et al., 2019). Besides, conferring to social exchange theory, Yin (2018) stated that the higher the degree of justice in an organization, the higher the psychological expectation of active work engagement among employees. Accordingly, employees can indicate such an exchange by performing tasks with greater dedication and being engaged in their work. In addition, recent studies on public hospital nurses in China, Zhu et al. (2015) showed that OJ has a significant correlation with WE. Besides, studies have shown that the relation between the experience of OJ and the actions of the organizational participants relates to WE (Bakker et al., 2007; Sharma and Yadav, 2017; Sharma and Kumra, 2020). Further, Kumasey et al. (2021) exposed that DJ and PJ were important organizational components and positively connected to WE.

DJ has been described as a major determinant of employee engagement (Gupta and Kumar, 2015; Soyer et al., 2021). In the same way, Sharma and Kumra (2020) found that DJ was positively related to job engagement. Besides, the DJ played a vital role in shaping the WE in the healthcare sector in Turkey (Özer et al., 2017). PJ is a major operational factor that influences the result distribution (Willison et al., 2018) and has a positive impact on WE workers (Kumasey et al., 2021; Özer et al., 2017), and is thus perceived to be a core component of a service company where there is a greater probability of work burn-out and turnover intention. In addition, based on the point of view of social exchange theory (Blau, 1964), there is a positive correlation between PJ and WE in organizations (Majumdar and Kumar, 2021). In particular, Özer et al. (2017) argued that PJ has the greatest effect on WE workers in contrast to other justice dimensions. Interactional justice (IJ) (e.g., Int.J and Inf.J) was favorably linked to WE (Stankevičiūtė and Savanevičienė, 2021). Similarly, Özer et al. (2017) observed in healthcare workers in Turkey that IJ was linked to WE and had a modest influence on WE. In addition, workers invest in their jobs more mentally, cognitively, and behaviorally and display a higher degree of commitment and loyalty to their job as they believe that Inf.J is present in their organizations (Gupta and Kumar, 2015; Soyer et al., 2021). Employees are thus supposed to engage in more constructive actions only at a stage when they perceive that their efforts would result in outcomes, they hoped for it. Simply, SET (Blau, 1964) could be the most appropriate theory to explain the relationship between interactional justice (IJ) (e.g., Int.J and Inf.J) and WE. This research thus assumes a positive association between the sense of OJ and WE of faculty members, and we project hypotheses accordingly.

H1: Distributive justice is positively related to work engagement.
H2: Procedural justice is positively related to work engagement.
H3: Interactional justice is positively related to work engagement.
H4: Informational justice is positively related to work engagement.

### 2.2 Work engagement and organizational citizenship behavior

WE is one of the essential devices that influences a varied range of attitudinal and behavioral outcomes in the workplace (Han et al., 2021). These outcomes of WE are employee job satisfaction (Tuin et al., 2021; Zaabi et al., 2016), commitment to the organization (Ahmad and Gao, 2018), reduces intention to quit (Cao and Chen, 2021; Saks, 2006), high levels of creativity (Bakker et al., 2014), task performance (Bakker and Albrecht, 2018; Gupta and Shukla, 2018), and OCB (Bakker and Albrecht, 2018; Ismael et al., 2021). In addition, more active workers in the workplace have a higher degree of OCB, which consequently enriches corporate success and increases productivity and efficiency (Organ, 1988; Zaabi et al., 2016). In addition, the exhibition of OCBs in companies is open to the individual preference of workers, and it is not punishable to exclude these behaviors (Rosario Núñez et al., 2020), so demonstration of these behaviors cannot be made mandatory (Turek, 2020).

Organ (1988) introduced five dimensions of OCB: altruism, courtesy, conscientiousness, civic virtue, and sportsmanship. The first component, altruism, encompasses spontaneous activities to assist other individuals with their jobs or resolve an organization-related issue. The second, courtesy, refers to attempts to avoid work-related conflicts with others. The third is conscientiousness, defined as discretionary action that goes above and beyond the role's requirements. The fourth, civic virtue, denotes a desire to responsibly engage in the organization's activities. The final component, sportsmanship, entails demonstrating patience in less-than-ideal conditions to avoid receiving complaints (Hanh Tran and Choi, 2019; Zúñiga et al., 2022). However, previous studies indicate that there are two distinct dimensions of OCB depending on the anticipated behavioral target, these are (i) *OCBI—OCB that are directed towards individual*s and/or groups in an organizational setting and (ii) *OCBO—OCB directed towards the organization* (Ashfaq and Hamid, 2020; Lee and Allen, 2002; Somech and Khotaba, 2017; Williams and Anderson, 1991). However, in this study, we are interested in only these two dimensions: OCBI and OCBO. We have chosen these two OCB dimensions to focus on specific beneficiaries (Williams and Anderson, 1991). Moreover, OCBI and OCBO include all five dimensions previously described (Ma et al., 2022). OCBIs are practices aimed at supporting people or coworkers within the organizational context (Majeed and Jamshed, 2021), which involve altruism and courtesy (Donia et al., 2018). Also, OCBOs include citizenship activities that influence and support the whole organization, consisting of behaviors such as conscientiousness, sportsmanship, and civic virtue (Memon et al., 2017; Organ, 1988; Rahman and Karim, 2019). Studies have shown that WE is connected to OCB, as a dedicated employee devotes him/her completely and is more excited about working beyond their officially pre-defined roles and thus participates in other actions that include OCB (Lyu et al., 2016; Matta et al., 2015; Thakre and Mathew, 2020). Consequently, when an engaged faculty member shows constructive behavior that supports his or her colleges or organization, in exchange, such beneficiaries are likely to reciprocate similar behavior in order to recognize his or her prior behavior. In addition, previous researchers suggested the existence of a positive association between WE and OCB (Abed and Elewa, 2016; Ismael et al., 2021; Saks, 2006). This research, therefore, assumes a constructive relationship between the faculty's engagement and OCB in their job.

H5: Work engagement is positively related to OCB directed towards the individual.
H6: Work engagement is positively related to OCB directed towards the organization.

### 2.3 The mediating role of work engagement

OJ reflects how employees view fairness at work and have a strong influence on employee behavior (Sharma and Kumra, 2020; Stamenkovic et al., 2018) and shape employee attitudes (Chen and Khuangga, 2021). OJ is the most studied field in terms of the OCB context (Colquitt et al., 2001). In addition, several experiments have been performed based on the interaction between the perception of OJ and OCB (Ajlouni et al., 2021; Jnaneswar and Ranjit, 2021; Lim and Loosemore, 2017). Organ (1988) has revealed that perceived OJ is one of the crucial predictors of OCB. In addition, workers will exhibit extra-role behaviors (OCB) as they feel that the policies and procedures of their respective organizations are ethical and equal (Donglong et al., 2020; Khaola and Rambe, 2020). Besides, WE is associated with OCB because committed workers are dedicated to working beyond their job roles that include OCB (Bakker and Albrecht, 2018; Ismael et al., 2021; Lyu et al., 2016). Further, WE could play its role as a mediator between numerous job resources and employees' outcomes (Aboramadan and Dahleez, 2020), and the Job Demand-Resource model offered the requisite guidance on the use of WE as a mediator (Al-Shbiel et al., 2018). In particular, expressed that job resources encourage learning and development, growth and increase employee WE; in response, workers will show positive job outcomes (Lesener et al., 2020). Thus, job resources (e.g. OJ) have the potential to increase WE and is linked to the positive organizational consequences (Piotrowski et al., 2021; Schaufeli et al., 2009). Aboramadan and Dahleez (2020) illustrated that WE mediated the relationship between job resources and OCB. Besides, previous researches provide evidence that WE is a significant predictor of OCB (Abed and Elewa, 2016; Bakker and Albrecht, 2018; Ismael et al., 2021; Saks, 2006). Thus, it can be realized that effective implementation of OJ would result in be a higher employee WE, and consequently would result in higher degrees of devotion and extra-role performances (OCBs).

To be more specific, DJ is a type of OJ that focuses on employees' perceptions of receiving a fair share of desirable work-related outcomes. When same-level faculties are paid differently, organization members may feel that there is no distributive justice, which can lead to poor performance, cooperation, and work quality, as well as unethical employee behavior. However, applying the viewpoint of the social exchange theory (Blau, 1964), when members of an organization believe they are being treated fairly, they are more likely to engage in their work, and this is likely to lead to extra-role behaviors (Donglong et al., 2020). So, DJ has been identified as a significant driver of WE (Soyer et al., 2021), whereas OCB is one of the results of WE (Ismael et al., 2021). Therefore, when faculties find an equal distribution of resources, they might be more engaged in their lectures, assessing students' performance and conducting research. In doing so, they might show volunteer concern for the university's performance (OCBO), students' performance, and co-researchers (OCBI).

PJ is more directly tied to an organization's evaluation and typically concerns its procedures for making a decision. Faculties' commitment to and satisfaction with the university and their engagement might suffer if they believe the decision-making process is not fair and just. However, relying on the social exchange theory, when organizations have just and fair decision-making, faculties show commitment and, in return, engage more in their work (Blau, 1964; Majumdar and Kumar, 2021). Therefore, in university setting, faculty members who are highly engaged in their profession are more likely to acquire more dedication to the institution and voluntary contribute more to university (OCBO) and its people (OCBI).

Int.J is about superiors treating subordinates with respect and kindness (Sharma and Kumra, 2020). Those that believe Int.J is present in their organizations put more mental, cognitive, and behavioral effort into their jobs (Soyer et al., 2021). From the social exchange view, when someone is treated well, they will demonstrate the same concern for the person who treated them well. When the authorities behave well toward the faculties in a university setting, it may positively affect them (Blau, 1964). There is a chance that they will become more concerned about the university's performance and more involved in their jobs (Stankevičiūtė and Savanevičienė, 2021), and show a higher level of concern and volunteer cooperative behavior (OCB) with the university authorities, students, and staff.

Inf.J requires people to be involved in decision-making and to be informed completely and timely about the process (Charoensap et al., 2019; Schumacher et al., 2020). Higher Inf.J leads to more behavioral and cognitive engagement with work, including increased commitment and motivation, pride in work (Chandani et al., 2016), and then OCB (Lönnqvist et al., 2022). According to social exchange theory, employees' perceptions of getting sufficient information resources lessen their ambiguity about work-related issues, hence increasing their satisfaction (Hanh Tran and Choi, 2019), and job satisfaction is the key driver of work engagement (Garg et al., 2018) and which motivates them to reciprocate with the organization by exhibiting OCB (Hanh Tran and Choi, 2019). Founded on the above justifications, the subsequent hypotheses were articulated:

H7: WE mediates the relationship between DJ and OCBI
H8: WE mediates the relationship between PJ and OCBI
H9: WE mediates the relationship between Int.J and OCBI
H10: WE mediates the relationship between Inf.J and OCBI
H11: WE mediates the relationship between DJ and OCBO
H12: WE mediates the relationship between PJ and OCBO
H13: WE mediates the relationship between Int.J and OCBO
H14: WE mediates the relationship between Inf.J and OCBO

**Figure 1:**
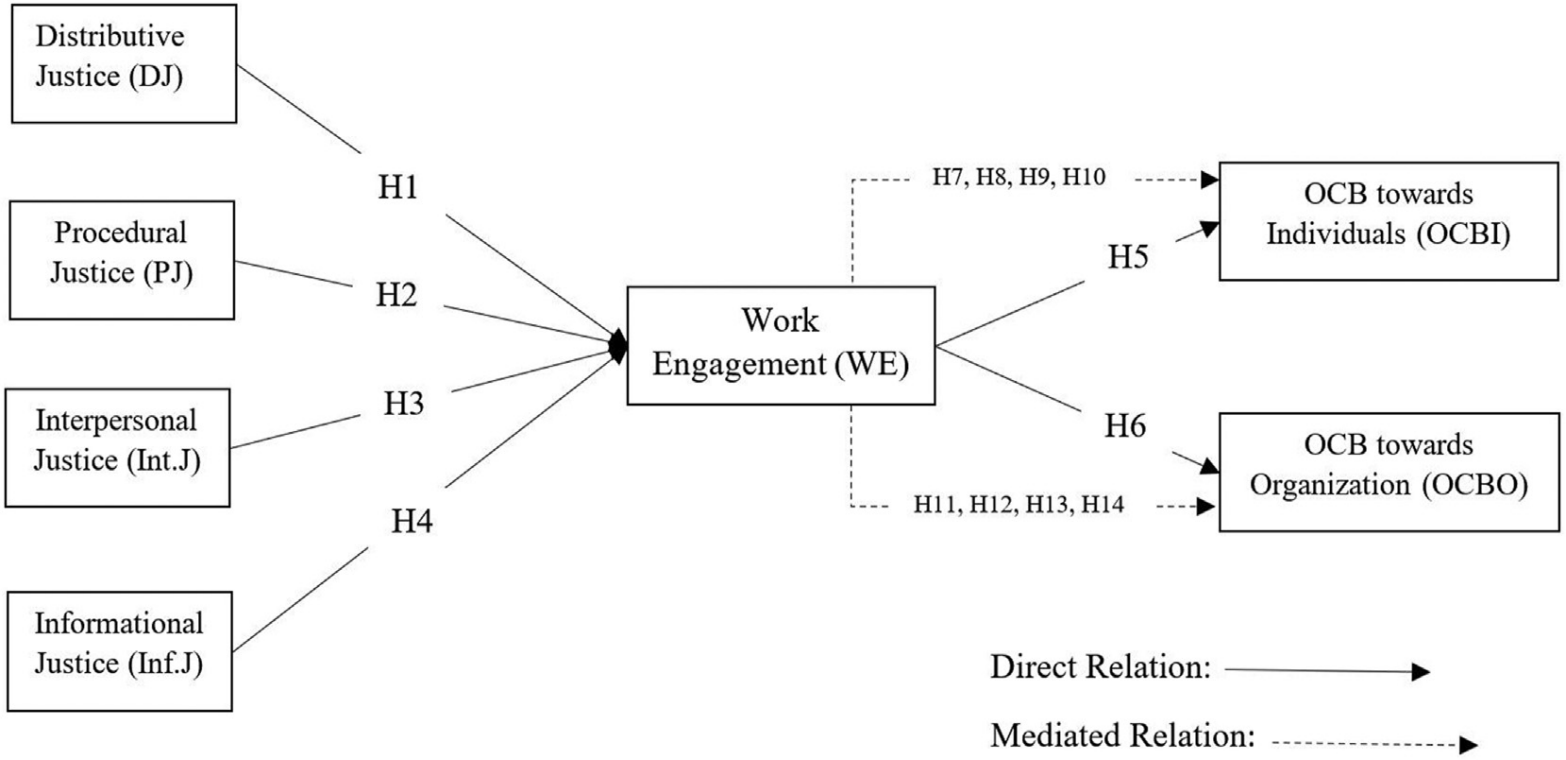
Conceptual framework and hypotheses.

The conceptual framework and the research hypotheses are available in Figure 1.

## 3 Research methodology

### 3.1 Population and sampling

All permanent faculty members in private universities in Bangladesh, regardless of their ranks, are included in the target population. According to 47^th^ Annual Report 2020, the total number of faculty members in all Bangladeshi universities is 30703 (male = 21661 and female = 9042). Among them, 15277 faculty members are serving in private universities (Bangladesh University Grant Commission, 2021). However, the current study purposively selected ten private universities operating in Dhaka city. A purposive sample is one whose features have been defined for a specific reason that is relevant to the study's objectives (Andrade, 2021). Dhaka, the capital of Bangladesh, was chosen for data collection because it is the capital of Bangladesh and includes two city corporations, Dhaka South and Dhaka North City Corporation, where most of the private universities are located. However, we purposively selected these ten private universities based on three criteria: i. They have a permanent campus; ii. They have more than 200 permanent faculty members; and iii. The Bangladesh University Grants Commission has not filed any allegations against this institution, and it is not under observation. Moreover, recently, national newspapers have regularly published reports on injustice and unfair activities, the autocracy of the chairman and board of trustees, and the poor performance of these universities. Besides, other concerns were convenience in terms of time, personal contact, accessibility, and budget. The total number of faculty members at these universities was 2798 (Bangladesh University Grant Commission, 2021). The individual samples for the study were selected based on the researcher's professional and personal network. Thus, we nominated representatives (faculties) from the selected universities based on personal relationships. The faculty members who had been nominated disseminated the questionnaire to their known contacts. However, when the researchers first considered systematic sampling, they were unable to get an accurate list of faculty members and failed to proceed. Although a few institutions' websites include a faculty list, the information is not updated regularly and can be misleading. For example, faculty on study leave and faculty who have left the organization were not updated. Therefore, the current research utilized a convenience sampling technique to collect data from the respondents. Though there are a few limitations to generalizing the result, social science research is heavily dominated by convenience sampling (Winton and Sabol, 2021). A total of 320 questionnaires was circulated mentioning one week to fill it out and mail using the self-addressed envelope submitted to them while the questionnaire was administered. We received 153 completed questionnaires where response rate is 47.81% and found 121 of them are useable. Therefore, the sample size is n = 121. Despite there is no precise rule of thumb, Hair et al. (2014) emphasized using 110 to 150 responses for fitting structural equation modelling, provided that the data has no issue with normality, missing value and homogeneity. Thus, the use of 121 responses with the absence of abnormality, outlier, missing value and heterogeneity of responses did not prevent us to advance for further investigation (Fan et al., 2019).

### 3.2 Research instrument

To serve the research intentions, relevant scales were adopted to confirm ample measurement of each intentional variable. The participants must respond to all items using a 5-point Likert scale, that ranges from “1 = strongly disagree” to “5 = strongly agree”.

#### 3.2.1 Organizational justice (OJ)

To measure the existence of OJ, the researchers adopted 20 items on four specific dimensions of OJ, named PJ, DJ, Int.J and Inf.J. To measure DJ and PJ, the present study adopts five items for DJ as well as six items for PJ from the scale established by Niehoff and Moorman (1993). Besides, four items for Int.J and five items for Inf.J has been taken from the scale developed to measure OJ by Colquitt (2001). To avoid the difficulty and to make it more credible to the respondents, a couple of wording changes were made to the items. Sample items include, *“Generally, the rewards I receive here are quite fair” and “My supervisor's explanations regarding the procedures are reasonable”.* The reliability of Cronbach's alpha for DJ and PJ were above 0.70 in the study of Niehoff and Moorman (1993). Additionally, the reliability of Cronbach's alpha for Int.J was 0.79 and for Inf.J was 0.79 in the study of Colquitt (2001).

#### 3.2.2 Work engagement (WE)

To measure WE the researchers of the current study adopted the “nine-item short Utrecht Work Engagement Scale-UWES” developed by Schaufeli et al. (2006) consisting of dimensions named vigor, absorption, and dedication. However, the current investigation uses this scale as unidimensional. Sample items contain “*At my job, I feel strong and vigorous*” and *“I am enthusiastic about my job*”. Besides, the reliability of Cronbach's alpha of these items' values ranged between .70 and .80 for 23% cases and it exceeded .80 for 67% cases conducted in different countries.

#### 3.2.3 5Organizational citizenship behavior

To evaluate OCB the current investigation adapted the OCB Scale (OCBS) consisting of sixteen items established by Lee and Allen (2002)**.** The OCBS is a widely used instruments, which is employed to assess OCB towards individuals (OCBI) and OCB toward the organization (OCBO). Sample items of the scale are *“I show genuine concern and courtesy toward coworkers, even under the most trying business or personal situations”,* and *“I offer ideas to improve the functioning of the organization”.* This scale is evident in many recognized studies (Harris et al., 2018; Jin et al., 2018; Lavy and Littman-Ovadia, 2017; Ong et al., 2018; Saks, 2006). Additionally, this scale is still relevant for measuring the existence of OCB among academics, as it was adopted in several studies, for example (Adil et al., 2021; Ahmad et al., 2019; Al-Mahdy et al., 2021; Atta and Khan, 2021; Butt et al., 2020; Cheung and Lun, 2015; Dirican and Erdil, 2016; Jin et al., 2018; Majeed and Jamshed, 2021; Umrani et al., 2019). Further, this scale, according to Panicker et al. (2018), might measure the five key components of OCB, such as conscientiousness, sportsmanship, civic virtue, courtesy, and altruism. To confirm the simplicity and to make it more plausible to the respondents a few wording changes were done to the items. In the study of Lee and Allen (2002) reliability was .83 (OCBI) and .88 (OCBO).

### 3.3 Ethical approval and respondents consent

Conducting social science research in Bangladesh is not subject to clearance under institutional practices and national legislation. Only sensitive scientific and medical experiments, like animal trials, require ethical approval here. However, the study was voluntary, and the author got permission and consent from the people who took part in it to share their data with the public.

### 3.4 Response & method bias

The researchers explored a variety of measures to eliminate response bias. Maintaining subject anonymity or including confidentiality promises can be effective methods for controlling Social Desirability Bias (Larson, 2019). Developing a good study information coversheet and set of instructions is one of the easiest strategies to improve answer accuracy (Jordan and Troth, 2020). First, the researchers assured the faculty members that their identities would be kept private in a written statement in the questionnaire's cover letter. It was declared that the research findings would be reported as a general industry trend rather than from the point of view of individual universities, which encouraged them to answer the questions accurately and confidently without fear of their identities being leaked. As a result, respondents were allowed to openly express their opinions regarding their organization's actions in terms of justice in the questionnaire (Jordan and Troth, 2020; Uddin et al., 2020). Besides, Harman's single factor test was conducted to identify any common method bias (Podsakoff et al., 2003). Further, evaluating the variance inflation factor (VIF) is another measure to check that the results of multiple linear regressions are free from common method bias (Kock, 2015). The calculated result of Harman's single factor test and VIF are available and discussed in the analysis and findings section. Moreover, the correlation matrix was calculated and is shown in Table III. The maximum correlation was 0.698, which is less than the 0.80 thresholds for any two constructs (Jordan and Troth, 2020; Tehseen et al., 2017).

### 3.5 Data preparation and analysis techniques

The descriptive statistical analysis was performed using SPSS version 25. Additionally, to test hypothesis and proposed conceptual model, SmartPLS3 was used to evaluate both the measurement and the structural model using partial least squares (PLS) path modeling. To test hypotheses and conduct analyses, we used partial least squares structural equation modeling (PLS-SEM). Co-variance-based SEM (CB-SEM) and Partial Least Square SEM (PLS-SEM) are two available techniques to estimate SEM (Astrachan et al., 2014). While PLS-SEM focuses on explaining the variance in endogenous variables, CB-SEM seeks to estimate the covariance matrix without focusing on explaining variance (Hair et al., 2017). Ringle et al. (2020) noted that PLS-SEM, a powerful multivariate analytic method, is frequently used by HRM scholars. For several reasons, PLS-SEM is preferable to CB-SEM. The CB-based SEM is used to assess an existing theory, while the PLS-SEM is used to build a theory and make a prediction. Second, although CB-SEM analyses solely reflecting interactions, PLS-SEM may research both formative and reflective interactions (Hair et al., 2017). Richter et al. (2016) say that PLS-SEM can handle complex models with a lot of indicators and constructs, deal with small sample sizes with care, and give meaningful latent variable scores that can be used in other studies.

## 4 Analysis and findings

### 4.1 Respondents profile

The demographic characteristics of the participants (n = 121) is reported as follows: among the faculties 44.6% (n = 54) were female and 55.4 % (n = 67) were male. 83.5% were married and 16.5% were unmarried. Most of the respondents, specifically 52.9 % (n = 64) were from 31-40 years and 31.4% (n = 38) were from below 30 years of age. Here, 43.8% (n = 53) were Assistant Professors, 33.1% (n = 40) were young Lecturers and the remaining 23.1% were from Professors and other designations. Most of the faculty members who responded completed only Post-graduation or Master's degree 86.0% (n = 104) and only 14.0% (n = 17) had their higher educational background (e.g. MPhil, PhD and Post-Doctoral or specialized degrees). In contrast to Western and European countries, a Master's or Post-graduate degree is adequate to join as a faculty member in Bangladesh. Additionally, Ph.D. or Post-Doctoral degrees are rarely required to be promoted to higher positions in Bangladesh. Rather than that, with few exceptions, promotion to a higher position is largely determined by length of service. In case of their tenure of service life in the current university, 50.4% (n = 61) were from 0-5 years of service, 28.9% (n = 35) were from 6-10 years and the remaining 20.7% (n = 25) has completed 15 years of service.

### 4.2 Measurement model

We assessed convergent validity considering the loadings of the items of the constructs and Average Variance Extracted (AVE). Initially, DJ and OCBI had AVEs lower than the criterion .50. To get AVEs above the cut-off point .50 and to get significant items loadings above the cut-off value .50 one item from DJ, three items OCBI and one item from OCBO have been deleted from the model as suggested by Hair et al. (2010), available in Table I. Then, the measurement constructs used in this investigation confirmed enough convergent validity as the AVEs of all the constructs was higher than .50 (Hair et al., 2019; Henseler et al., 2014). The Composite Reliability (CR) for the constructs of this research ranged between 0.830 to 0.915 and much better compared to the advocated cut-off value of .70 (Hair et al., 2019). Besides, CA— Cronbach's Alpha values for the constructs were higher than .728 which specified an ample amount of reliability as the cut off level is 0.70. Further, Dijkstra–Henseler's indicator rho_A coefficients were above the standard value of 0.70 (Hair et al., 2019). Hence, reliability criteria had been met for both at the item and construct level.

**Table I:** Internal consistency reliability and convergent validity.

Constructs	Measurement Items	Outer Loadings	CA α	rho_A	CR	AVE
Distributive Justice (DJ)	DJ1	.695	.728	.731	.830	.551
	DJ2	.738				
	DJ3	.829				
	DJ4	.698				
Procedural Justice (PJ)	PJ1	.721				
	PJ2	.883				
	PJ3	.896				
	PJ4	.860				
	PJ5	.784				
	PJ6	.736				
Interpersonal Justice (Int.J)	Int.J1	.837	.876	.883	.915	.729
	Int.J2	.871				
	Int.J3	.894				
	Int.J4	.811				
Informational Justice (Inf.J)	Inf.J1	.701	.856	.873	.897	.637
	Inf.J2	.834				
	Inf.J3	.862				
	Inf.J4	.859				
	Inf.J5	.719				
Work Engagement (WE)	WE1	.624	.888	.895	.910	.532
	WE2	.774				
	WE3	.755				
	WE4	.797				
	WE5	.788				
	WE6	.722				
	WE7	.743				
	WE8	.743				
	WE9	.589				
OCB towards Individual (OCBI)	OCBI1	.814	.751	.766	.834	.502
	OCBI2	.725				
	OCBI3	.659				
	OCBI5	.666				
	OCBI7	.667				
OCB towards Organization (OCBO)	OCBO2	.654	.859	.868	.892	.543
	OCBO3	.708				
	OCBO4	.755				
	OCBO5	.707				
	OCBO6	.826				
	OCBO7	.695				
	OCBO8	.799				

**Note:** Items DJ5, OCBI4, OCBI6, OCBI8 and OCBO1 had been deleted for better AVEs.

After evaluating convergent validity, we examined the discriminant validity— the extent to which constructs are discrete (Hair et al., 2010). In SEM, the heterotrait-monotrait ratio of correlations (HTMT) is suggested by (Henseler et al., 2014) as a relatively and consistently new mechanism to evaluate discriminant validity. Where, in a more strict approach to use HTMT, a threshold value of 0.85 as suggested in (Hair et al., 2019; Kline, 2015), while other SEM academic researchers indorsed a more flexible value of 0.90 (Gold et al., 2001; Hair et al., 2019). Here, the constructs meet the threshold according to a more strict approach as HTMT <0.85 (Kline, 2015), available in Table II. Additionally, to inspect the discriminant validity we evaluated items cross-loadings and inter-correlations of the constructs (see Table III) and found satisfactory. In a nutshell, all the latent constructs included in this investigation disclosed ample reliability and validity to proceed to assess the structural model.

**Table II:** Descriptive statistics for constructs and assessing discriminant validity using HTMT.

Constructs	Mean	Std. Dev.	1	2	3	4	5	6	7
1. Distributive Justice (DJ)	3.564	0.726							
2. Informational Justice (Inf.J)	3.709	0.721	.319						
3. Interpersonal Justice (Int.J)	3.940	0.772	.380	.807					
4. OCB towards Individual (OCBI)	4.150	0.498	.478	.313	.354				
5. OCB towards Organization (OCBO)	4.282	0.548	.376	.472	.537	.626			
6. Procedural Justice (PJ)	3.156	0.910	.187	.696	.620	.190	.269		
7. Work Engagement (WE)	4.059	0.637	.499	.673	.691	.525	.612	.455	

Note: Discriminant Validity is established at HTMT_0.85_.

**Table III:** Correlations among the constructs.

Constructs	1	2	3	4	5	6	7
1. Distributive Justice (DJ)	***.742***						
2. Informational Justice (Inf.J)	.252	***.798***					
3. Interpersonal Justice (Int.J)	.315	.698	***.854***				
4. OCB towards Individual (OCBI)	.380	.255	.303	***.709***			
5. OCB towards Organization (OCBO)	.303	.404	.473	.505	***.737***		
6. Procedural Justice (PJ)	.104	.600	.559	.123	.243	***.816***	
7. Work Engagement (WE)	.411	.603	.624	.441	.540	.417	***.729***

**Note:** Italic values on the diagonal represent the square root of AVE & the other entries represent the correlations.

### 4.3 The structural model

The structural model inspects the causal relationships among the latent constructs in a model (Memon et al., 2017). To avoid possible biases in regression results researchers must address the collinearity issue first and SEM researchers recommended using VIF— Variance Inflation Factor to examine the collinearity (Memon et al., 2017). Besides, the model can be regarded free of common method bias if all VIFs from a comprehensive collinearity test are equal to or less than 3.3 (Kock, 2015). In our study, the VIF values reported for each latent construct were below 3.3, thus, the study was unaffected by collinearity issues.

#### 4.3.1 Direct relations

**Figure 2:**
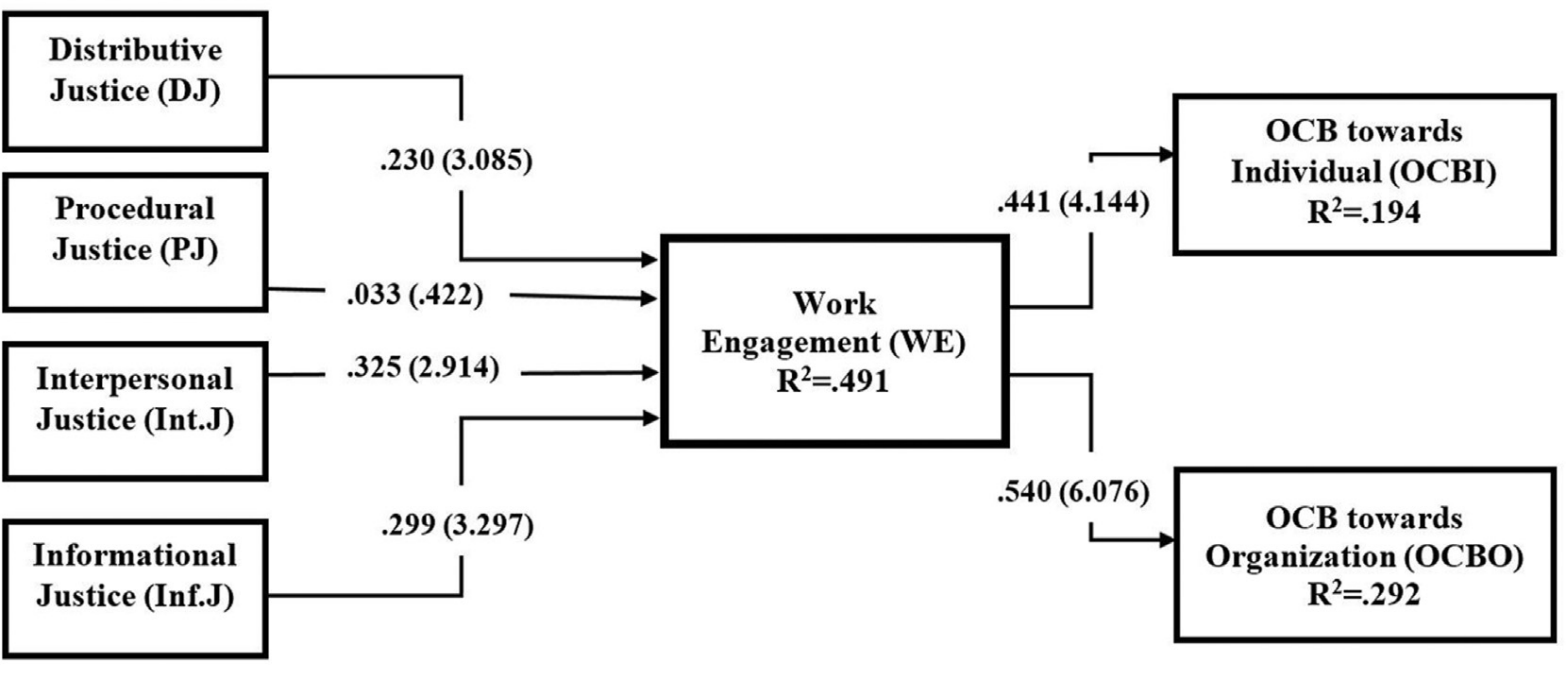
Structural model representing R^2^, beta coefficient and t-values for path coefficients.

To inspect the statistical magnitude of the path coefficients present study employed the bootstrapping technique (resampling = 5000, minimum) (Hair et al., 2017). Here, we examine the links between independent and dependent variables by assessing the t-values and significance level at 0.05 (p < .05). Besides, we considered the Bias Corrected Confidence Intervals (BCI LL-UL) to report the significance of the hypothesized relationships (Hair et al., 2019). Among direct relationships in Table IV and Figure 2, except for hypothesis H_2_ (PJ → WE), we noticed that other hypotheses are statistically significant.

**Table IV:** The result of the structural model assessment for direct relations.

H	Relations	Std Beta	Std Error	T values	P Values	BCI LL	BCI UL	f^2^	VIF	Decision
1	DJ → WE	.230	.074	3.085	.001	.097	.344	.092	1.128	Supported
2	PJ → WE	.032	.075	.422	.336	-.089	.153	.001	1.685	Not Supported
3	Int.J → WE	.325	.112	2.914	.002	.137	.507	.095	2.182	Supported
4	Inf.J → WE	.299	.091	3.297	.000	.151	.447	.078	2.241	Supported
5	WE → OCBI	.441	.106	4.144	.000	.238	.590	.241	1.000	Supported
6	WE → OCBO	.540	.089	6.076	.000	.360	.665	.412	1.000	Supported

Note: BCI LL- Confidence Intervals Bias Corrected Lower Limit.

BCI UL- Confidence Intervals Bias Corrected Upper Limit.

Here, DJ (H1: DJ → WE, β = 0.230, t = 3.085, p < 0.05, BCI LL: 0.097, UL: 0. 344) was significantly and positively related to WE. PJ (H2: PJ → WE, β = 0.032, t = 0.422, p > 0.05, BCI LL: -.089, UL: 0.153) was not significantly related to WE. Besides, Int.J (H3: Int.J → WE, β = 0.325, t = 2.914, p < 0.05, BCI LL: 0.137, UL: 0.507) was significantly and positively related to WE. Likewise, Inf.J (H4: Inf.J → WE, β = 0.299, t = 3.297, p < 0.05, BCI LL: 0.151, UL: 0.447) was significantly and positively related to WE. Further, WE (H5: WE → OCBI, β = 0.441, t = 4.144, p < 0.05, BCI LL: 0.238, UL: 0.590) was significant and positively linked to OCBI. Furthermore, WE (H6: WE → OCBO, β = 0.540, t = 6.076, p < 0.05, BCI LL: 0.360, UL: 0.665) was significantly and positively related to OCBO.

#### 4.3.2 Specific indirect effects

The mediating effect of WE has been checked to find whether it works as mediating factor between the relationships DJ, PJ, Int.J, Inf.J and OCB. Here, in case of OCBI, WE (H7: DJ → WE → OCBI, β = 0.101, t = 2.237, p < 0.05, BCI LL: 0.038, UL: 0.181) mediated the association between DJ and OCBI. But WE (H8: PJ → WE → OCBI, β = 0.014, t = .394, p > 0.05, BCI LL: -0.040, UL: 0.074) failed to mediate the link between DJ and OCBI. Besides, WE (H9: Int.J → WE → OCBI, β = 0.143, t = 2.375, p < 0.05, BCI LL: 0.059, UL: 0.256) mediated between the DJ and OCBI relation. Similarly, WE (H10: Inf.J → WE → OCBI, β = 0.143, t = 2.540, p < 0.05, BCI LL: 0.056, UL: 0.225) mediated between the DJ and OCBI relationship. On the other hand, in terms of OCBO, WE (H11: DJ → WE → OCBO, β = 0.124, t = 2.790, p < 0.05, BCI LL: 0.053, UL: 0.196) mediated the relationship between DJ and OCBO. However, WE is not a mediator (H12: PJ → WE → OCBI, β = 0.017, t = 0.417, p > 0.05, BCI LL: -0.051, UL: 0.082) between DJ and OCBO relationship. Further, WE (H13: Int.J → WE → OCBI, β = 0.176, t = 2.425, p < 0.05, BCI LL: 0.073, UL: 0.312) mediated the association between DJ and OCBO and finally, we found WE as a mediator (H13: Inf.J → WE → OCBI, β = 0.162, t = 2.791, p < 0.05, BCI LL: 0.075, UL: 0.266) between DJ and OCBO relationship (available in Table V).

**Table V:** The result of the structural model assessment for Specific Indirect Effects.

H	Relations	Std Beta	Std Error	T value	P Value	BCI LL	BCI UL	Decision
7	DJ → WE → OCBI	.101	.045	2.237	.013	.038	.181	Supported
8	PJ → WE → OCBI	.014	.036	.394	.347	-.040	.074	Not Supported
9	Int.J → WE → OCBI	.143	.060	2.375	.009	.059	.256	Supported
10	Inf.J→ WE → OCBI	.132	.052	2.540	.006	.056	.225	Supported
11	DJ → WE → OCBO	.124	.044	2.790	.003	.053	.196	Supported
12	PJ → WE → OCBO	.017	.041	.417	.338	-.051	.082	Not Supported
13	Int.J → WE → OCBO	.176	.073	2.425	.008	.073	.312	Supported
14	Inf.J→ WE → OCBO	.162	.058	2.791	.003	.075	.266	Supported

To report the magnitude of the relationships, Hair et al. (2017) suggested evaluating the R^2^ and f^2^. Here, R^2^— coefficient of determination denotes the predictive capability of the exogenous construct(s) to determine the variability in the endogenous variable in a model (Memon et al., 2017). Hair et al. (2019) and Henseler et al. (2009) suggested that the R^2^ values of 0.75, 0.50, and 0.25 can be referred to as substantial, moderate, and weak predictive power. In our study, we found R^2^ (WE-0. .491) which indicated that independent variables DJ, PJ, Inf.J, and Int.J can explain 49.1% variability of WE. Besides, R^2^ (OCBI-0.194) indicated that WE can explain 19.4% variability in OCBI as well as R^2^ (OCBO-.292) specified that 29.2% variability in OCBO can be explained by WE (see Figure 1). Therefore, DJ, PJ, Inf.J, and Int.J had moderate to a substantial level of predictive power to predict WE; and WE had a moderate level of predictive power to predict OCBI and a weaker predictive power to predict OCBO.

Now, f^2^—effect size; the amount to which an exogenous variable contributes to the R^2^ of the endogenous variable (Cohen, 1988). introduced a criterion mentioning that f^2^ values above 0.35, 0.15, and 0.02 illustrate a large, medium, and small effect size and if f^2^ < 0.02, there is no effect. Following Cohen in our research, only the relationship PJ → WE (H2: f^2^ < 0.02) did not affect the R^2^ of WE. However, for other relationships, this research found a small effect to large effect, available in Table VI.

**Table VI:** The Results of R2, Q2 and f2.

Construct	(R^2^)	(Q^2^)	Effect Size (F^2^)		
			WE	OCBI	OCBO
Distributive Justice (DJ)			.092- small effect		
Procedural Justice (PJ)			.001- no effect		
Interpersonal Justice (Int.J)			.095- small effect		
Informational Justice (Inf.J)			.078- small effect		
Work Engagement (WE)	.491	.248		.241- medium positive effect	.412 large positive effect
OCB towards Individual (OCBI)	.194	.078			
OCB towards Organization (OCBO)	.292	.135			

Chin et al. (2008) stated that Q^2^ works as a representative of out-of-sample prediction as well as in-sample predictive power for the endogenous construct, as mentioned in (Hair et al., 2017). According to Hair et al. (2017), "In the structural model, Q^2^ values larger than zero for a specific reflective endogenous latent variable indicate the path model's predictive relevance for a particular dependent construct" (p. 202). Memon et al. (2017) indicated that the values of Q^2^ were calculated through a blindfolding procedure using the construct's cross-validated redundancy values.

As shown in Table VI in this study, WE (Q^2^ = .248) indicated an acceptable predictive relevance of DJ, PJ, Int.J and Inf.J in predicting WE. Besides, OCBI (Q^2^ = .078), OCBO (Q^2^ = .135) specified a satisfactory predictive relevance of WE on OCBI and OCBO. The study used self-reported data. Thus, it might create an opportunity for common method variance (CMV). We conducted the Harman single-factor test and noticed that the single factor was accountable for only 25.61 per cent of the entire variance which was lower compared to the recommended of fifty per cent by (Podsakoff et al., 2003), and so, CMV was not present in this experiment.

## 5 Discussion on findings and implications

There are both practical and theoretical implications for this investigation. Here we discuss both managerial and theoretical contribution.

### 5.1 Managerial implication

The goal of this research was to see if there was a link between four aspects of OJ and different dimensions of OCB, as well as to see if WE might play a mediating role for faculty members at private universities in Bangladesh. The outcomes of the current investigation are as per the hypothesized relations. The study found a significantly positive relationship between DJ, and WE. This finding is related to the study by Gupta and Kumar (2015), Ghosh et al. (2014), and Gupta and Kumar (2012). Similarly, Int.J and Inf.J are positively related to WE which is similar to the study of Agarwal (2014). Remarkably, we found that PJ is not significantly related to WE which is the deviation of the studies by Agarwal (2014) and Gupta and Kumar (2015), and Özer et al. (2017). However, Saks (2006) also failed to establish a significant positive relationship between PJ and WE. Based on the corresponding lowest mean and higher standard deviation of ($\bar{\text{x}}$= 3.156, SD = 0.910), one possible explanation is that in most of the cases, Bangladeshi private universities demonstrated inconsistency in actions and decision-taking, and the approved processes were inaccurate, unethical, biased, and inappropriate. Furthermore, PJ entails listening to employee feedback and involving employee groups from a variety of work responsibilities and employment sectors in the decision-making process (Kim and Park, 2017), which is notably lacking due to the Chairman and Board of Trustees' authoritarian behavior (Sarkar and Hossain, 2019). Teachers perceived the organization's unfairness and did not respond positively by demonstrating positive engagement with the university. Basically, a social exchange connection says that a person who has received a service from another party is expected to express thanks when the opportunity comes up. Failure to express gratitude and appreciation may convey the impression that the individual is ungrateful and does not deserve the help (Blau, 1964; Cenkci et al., 2021).Thus, with exception to PJ, this research showed, in line with prior empirical investigations, that academics who had been treated fairly in distributing pay and awards, decision making and their contact with superiors had been more engaged in their job than those that were not fairly treated (Ghosh et al., 2014; Gupta and Kumar, 2012, 2015; Stankevičiūtė and Savanevičienė, 2021). From Table II, the mean scores for all the justice dimensions are between 3.156 to 3.940 on a 5-points Likert Scale. This means that the level of justice and fairness applied in private universities is considerably below the substantial level. In this study, faculty members could reduce their engagement to restore fairness. The university authorities might therefore concentrate on strengthening PJ, DJ, Inf.J, and Int.J related to faculty members.

The research, however, documented a substantial positive association between engagement and the dimensions of OCB. The findings are related to the previous studies conducted by (Abed and Elewa, 2016; Bakker and Albrecht, 2018; Runhaar et al., 2013; Saks, 2006). The results indicated that employees with high WE are more prone to exhibit OCBI and OCBO. Thus, an engaged faculty member might devote him/her completely and might be more excited about working beyond their officially pre-defined roles of teaching, research and administrative tasks and accordingly participate in other actions that include OCB (Lyu et al., 2016; Matta et al., 2015). Consequently, when an engaged faculty member exhibits constructive behavior that helps his or her institution, such recipients are likely to reciprocate such conduct in order to acknowledge their previous behavior (Runhaar et al., 2013). However, the mean scores of WE and OCB dimensions in this research range from 4.059 to 4.282 with minimal standard deviation indicate that there are acceptable levels of WE and OCBs among private university faculty members. Importantly, we found a positive association between the WE and OCB dimensions, providing us with a rationale to interpret that higher WE equals higher OCBs. These findings would contribute the private HEIs greatly. Since the work of teachers is highly individualistic and difficult to explain. The teacher's primary responsibilities are classroom teaching, research, and administrative work. Recently, research has become one of the significant indicators of university performance, but it depends highly on faculty volunteer initiatives. A greater level of OCB from the academic staff is likely to boost collaborative research work. The voluntary cooperation of senior and experienced faculty can help new researchers discover research problems and learn how to conduct research, operate research software, and use technologies. In teaching, voluntary and helpful behaviors of academics can play a big role in collaborating with students on solving exercises, providing additional examples, extending lectures outside of the syllabus but in relevant contexts, and sharing more course-related resources with students. These behaviors will produce quality students as potential marketable graduates. Empirically, a common criticism from the corporate sector in Bangladesh is that the Bangladeshi universities are not producing quality graduates ready for employment.

The administrative activities in Bangladeshi universities are very bureaucratic and slow. By cooperating with office staff, faculties can solve unusual issues by applying their theoretical expertise. In addition, faculties can assist with new students' admission processes, preparing class schedules, and preparing results and grade sheets. Therefore, minor but voluntary cooperation from faculties toward admin officers may solve performance issues. In addition, faculties can provide counseling, another volunteer role, to students to improve their mental health, especially in pandemics like COVID-19. In addition, they should cooperate with students in their career choices and guide new faculties toward their careers. Further, in Bangladesh, only a few universities initiate faculty development programs to orient faculty members. So senior faculties proactively orient new faculty members to improve their performance and the university's performance. Thus, OCB can be one of the most fruitful mechanisms to respond to the existing performance gap in the private education sector in Bangladesh.

Moreover, COVID-19 has made OCB more important for enhancing university performance. During COVID-19, the experienced and skilled faculties may cooperate with the inexperienced faculties to adopt online teaching technologies since adopting online teaching technologies has become a must. So, to compete globally, universities need faculty OCBs. Particularly in Bangladesh, where the private higher education sector is highly competitive and lagging in providing a higher quality of education. Furthermore, poor performance causes many private universities to close. As a result, these universities can improve performance by enhancing OCB.

Finally, the topmost finding of the current study is that faculty engagement was discovered to have an essential mediating role in the link between OJ and OCB. WE mediated the relationship between DJ, Int.J, Inf.J, and OCB dimensions- OCBI and OCBO. Therefore, WE serves as a mediator between DJ, Inf.J, and Int.J and OCB- OCBI and OCBO, implying that OJ dimensions can impact OCBI and OCBO directly or indirectly via employee engagement. This research, however, failed to establish WE as a mediator between PJ and OCB. Thus, OJ and WE play a great role in the promotion of OCB among faculty members. It will create positive energy among faculty members if they are treated fairly, which involves the distribution of outcomes, taking decisions that are indifferent to any member, sharing information based on which the decisions are made and offering clarifications for every decision made. They want to devote themselves to the universities on a physical, cognitive, and emotional level, and they are interested in working more better. Thus, if private higher education institutions could get their faculty more engaged, they might be able to make more OCBI and OCBO, which would lead to more productivity and performance.

### 5.2 Theoretical contribution

Theoretically, when people see unfairness, they generally modify their actions to reestablish equality by adjusting contribution to correspond with results, such as departing the office early, making fewer efforts or lowering their good attitude or contribution (Donglong et al., 2020). Empirically, faculty members strive to be more engaged in their job in exchange for justice and fairness (Stankevičiūtė and Savanevičienė, 2021). Furthermore, research has revealed a link between WE and OCB. Because an engaged employee is more excited about working beyond their formally stated obligations and is more committed to tasks, they are more likely to participate in OCB-related activities (Bakker and Albrecht, 2018; Geus et al., 2020; Lyu et al., 2016). Therefore, we detected an exchange connection between OJ dimensions to WE; and WE to OCB dimensions, which might be described by applying SET (Blau, 1964). As previously mentioned, few researchers have explored the role of WE as a mediator between the dimensions of the OJ and the dimensions of the OCB. The present study, therefore, adds to the available of knowledge and theory on the relation between OJ and OCB, OJ and WE, especially in Social Exchange Theory (Blau, 1964). To be specific, the role of WE in the OJ-OCB relationship. OJ reflects that faculty members experience fairness in their universities in terms of allocation of wealth and income, defined policies, relationships and exchange of information, and such perceived fairness produce a belief of positive self-image and self-distinctiveness that essentially serves as a strong engagement stimulus. It can be explained as a consequence of social relationship exchanges (Blau, 1964). In the same way, WE is linked to OCB because engaged faculties like to work above their defined duties, academically referred to as OCB. This connection is based on the Social Exchange Theory as well. When faculties are engaged in working, they are concerned about the people around them. At the same time, because they work for the university, they are more dedicated to it and voluntarily display discretionary behavior toward individuals and the campus. Faculties who are treated justly may demonstrate more work engagement and are more likely to reciprocate with discretionary behavior. Therefore, to get the faculties to operate on a voluntary basis for other faculties and universities without any incentive intent, the university administration and management should take the requisite steps to raise their level of engagement through the establishment of justice.

## 6 Limitations and further scope of the study

There are some drawbacks encountered in the generalization of the findings of this investigation. The sampling frame was restricted only to two-city corporations; thus, the sample somehow may not accurately represent the whole population. In future experiments, researchers may collect data from a wider sampling frame. In particular, a relatively small sample size was considered, limiting the research findings. For this reason, a larger sample size may be explored for future investigations. Social desirability is another issue that needs to be looked at more carefully in future studies because the study is directly linked to how the people in the study act and behave. Finally, the data used for this analysis are cross-sectional and hence may not signify the genuine context to some extent. Future research may consider careful designation of the questionnaire, language adjustments, multi-scaling and longitudinal studies may be carried out to avoid the issue. While this study has illustrated for the first time the existence of WE's mediating role between the dimensions of OJ and OCB, it deserves a more in-depth understanding of how these relationships function. Therefore, in order to avoid these shortcomings and to generalize the framework, in both profit and non-profit industrial settings as well as government and non-government organizational settings, additional conclusive research steps should be taken on the issue.

## 7 Conclusion

Organizational citizenship behavior (OCB), or favorable, natural, and discretionary extra-role behaviors of employees, significantly impacts desired job outcomes and institution efficiency. Service organizations, in particular higher education institutions, increasingly expect OCB from employees without directly rewarding them. Besides, it is evident that academicians' OCB has become a key performance metric for academic and institutional success. Empirically, organizational justice has been found to be a strong predictor of OCB. As evident in the literature, justice successfully generates employees' desire for extra-role conduct, particularly through a causative mechanism. In other words, organizational fairness does not automatically lead to OCB. Organizational commitment, trust, work happiness, and job commitment have all been explored as intervening factors between organizational justice and OCB. However, there is still a demand to uncover the "black box" in the justice —OCB interaction. Few studies have looked at the mediating role of work engagement in the link between justice and OCB across industries regarding the influence of OJ on employee attitudes and behavior. Therefore, we initiated this study to investigate the mediating role of work engagement between the four dimensions of justice and the OCB dimensions. Specifically, the mediating influence of work engagement in the relationship between justice and OCB in HEIs is not currently being investigated. It was decided to collect data from faculty members from private universities in Bangladesh to check the mediating relations. The PLS-SEM technique was used to evaluate the significance of the relationships. According to the experiment's findings, justice is favorably associated with WE. Besides, WE has a substantial relationship with OCB. Further, WE was a mediating factor in the link between the justice and OCB dimensions. The study findings were insufficient to firmly generalize the results due to several shortcomings. These include a small sampling frame and sample size, the cross-sectional nature of the study, and social desirability. Future studies should employ proactive initiatives to get rid of these limitations to produce more precise results. Although WE was discovered as a mediator between justice and OCB in this study, more research is needed to confirm these relationships. To prevent these problems, this model should be generalized in both profit and non-profit industrial contexts as well as government and non-government organizational settings. Therefore, the study gives crucial advice for organizations on how they can promote citizenship behavior by assuring fairness and engagement in the workplace. As a result, if the findings are put into practice, they may result in better employee outcomes and increased productivity at work.

## Declarations

### Author contribution statement

Md. H Asibur Rahman: Conceived and designed the experiments; Performed the experiments; Analyzed and interpreted the data; Wrote the paper.

Dewan Niamul Karim: Contributed reagents, materials, analysis tools or data; Wrote the paper.

### Funding statement

This research did not receive any specific grant from funding agencies in the public, commercial, or not-for-profit sectors.

### Data availability statement

Data will be made available on request.

### Declaration of interests statement

The authors declare no conflict of interest.

### Additional information

No additional information is available for this paper.
